# Are there differences in the growth adaptation processes of growing and mature organism models of short bowel syndrome?

**DOI:** 10.6061/clinics/2018/e499

**Published:** 2018-10-10

**Authors:** Ana Cristina Aoun Tannuri, Ítalo Geraldo Rotondo, Guilherme Garcia Barros, Victor Van Vaisberg, Cícero Mendes-Neto, Vitor Ribeiro Paes, Maria Cecilia Mendonça Coelho, Josiane Gonçalves, Suellen Serafini, Uenis Tannuri

**Affiliations:** Divisao de Cirurgia Pediatrica, Unidade Pediatrica de Transplante de Figado e Laboratorio de Pesquisa em Cirurgia Pediatrica (LIM 30), Faculdade de Medicina FMUSP, Universidade de Sao Paulo, Sao Paulo, SP, BR

**Keywords:** Short Bowel Syndrome, Ileocecal Resection, Intestinal Adaptation, Weanling Rats, Animal Model

## Abstract

**OBJECTIVES:**

The purpose of this study was to present an experimental model of short bowel syndrome (SBS) in weaning rats and to compare the adaptative mechanisms of the remaining bowel in weaning rats and adult animals by means of morphometric, histologic and molecular methods.

**METHODS:**

Twenty-four weaning rats were divided into 3 groups of 8 animals, one control group and two short bowel groups (euthanasia after 4 and 21 days), and were compared with similar adult groups. Morphometric evaluations of the animals and histopathological and molecular studies of the remaining bowel were performed.

**RESULTS:**

The weight of young rats increased after enterectomy, whereas that of adult rats decreased after enterectomy (*p*<0.0001). The ratio of intestinal length/body weight was significantly higher in weaning rats than in adults (*p*<0.002), showing that intestinal growth was more intense in weaning rats. Intestinal resection promoted increased thickness of the small bowel lamina propria (*p*=0.001) and reduced thickness of the colon lamina propria (*p*=0.04) in weaning rats relative to those in adults. In addition, intestinal resection promoted increased expression of the Bcl-xl gene (antiapoptotic) in adult animals compared with that in weaning rats (*p*=0.001).

**CONCLUSION:**

Morphometric, histological and molecular differences were shown in the adaptation processes of growing and mature organisms.

## INTRODUCTION

Short bowel syndrome (SBS) is a state of malnutrition and malabsorption that can result from massive bowel resection 1,2. In children, the most common causes of SBS are necrotizing enterocolitis, midgut volvulus and gastroschisis 3.

Although intestinal transplantation is a treatment option for this condition, recent data reinforce the role of comprehensive intestinal rehabilitation programs in promoting the native remaining bowel to acquire full absorptive capacity 4-6.

Therefore, the adaptability of the absorption surface of the remaining bowel segment is the main factor that decreases the malabsorption and malnutrition caused by SBS. “Intestinal adaptation starts within 24-48 hours after resection and includes structural and functional changes in the remaining bowel. The period of adaptation is approximately 15 days in rats, but it is much longer in humans 7,8.”

“The indicators of bowel adaptation in SBS have been reported as increased bowel and mucosal weight and changes in the DNA and protein content of the mucosa, villus height, and crypt depth 9,10.”

In regenerative processes, there is a balance between cellular proliferation and death. Apoptosis (programmed cell death) results from a balance between the expression of proapoptotic genes (such as Bax and Bak) and antiapoptotic genes (such as Bcl-2 and Bcl-xl) 11. In regenerative processes, such as intestinal adaptation, where proliferation must exceed cell death, there is greater tissue expression of antiapoptotic genes than of apoptotic genes 12.

Several experimental models utilizing adult rats have been described to elucidate the mechanisms of intestinal adaptations after massive intestinal resections. However, very few models have been developed in growing rats to elucidate pediatric SBS 13. In many other situations, it was proven that an immature organism's responses to insults are different than those of adults, and it is believed that children have a better prognosis than adults in SBS. Only one study has compared mucosal adaptation after massive bowel resection in weaning and adult rats, and the authors concluded that adaptative processes were more apparent in adult animals than in young animals 14. However, the bowel resection in this study involved the jejunum and not distal segments of the bowel. Therefore, it did not reflect the most common pediatric clinical condition, where extensive resections (mainly caused by necrotizing enterocolitis) generally involve the ileum and proximal colon 15.

Standardization of the same ileocecal resection model in weaning and adult rats with the aim of comparing the adaptative mechanisms between them would be valuable for elucidating specific histological and physiological changes after massive pediatric bowel resections and would consequently contribute to therapeutic and prognostic perspectives in children with SBS. Therefore, the objectives of the current study were to compare the adaptative mechanisms of the remaining bowel in weaning and adult rats using morphometric, histologic and molecular methods.

## MATERIALS AND METHODS

Twenty-four young Wistar rats (50-70 g body weight; 21 days old) and twenty-four adult Wistar rats (250-300 g body weight) were used. All applicable international, national, and/or institutional guidelines for the care and use of animals were followed. This study protocol was reviewed and approved by the Ethics Committee of our institution. The rats were maintained on a standard laboratory diet, and tap water was provided ad libitum throughout the experiment.

The animals were divided into young (Y) and adult (A) groups. In both groups, death and sample collection occurred at two times: one group was euthanized four days after bowel resection (Y1 and A1), and the other group was euthanized 21 days after resection (Y2 and A2). Controls of the same age and weight were used for both groups (YC and AC for Y and A, respectively).

### Surgical procedure

The rats were fasted for 12 hours prior to surgery but had free access to water. Following the induction of anesthesia by isoflurane inhalation, 50 mg/kg ketamine hydrochloride (Ketalar®, Eczacibasi, Istanbul, Turkey) was injected intramuscularly for long-term anesthesia and analgesia.

A 3-cm sagittal midline abdominal incision was performed, and the small intestine and colon were exposed. Intestinal length was measured in a standard fashion. Superior mesenteric vessels were ligated distally to the 5^th^ branches, and 60% of the small bowel, cecum, and appendix and 2 cm of the proximal colon were resected. The continuity of the remaining intestine was restored with end-to-end anastomosis with 8/0 polypropylene sutures (Prolene®, Ethicon) (Johnson do Brasil, São José dos Campos, SP, Brazil).

Following an injection of 10 mL of saline solution into the abdominal cavity, the abdomen was closed with 4/0 polypropylene sutures. The animals received 50% glucose and sodium supplementation in water on the first postoperative day.

### Euthanasia

After the corresponding time period for each group, euthanasia was performed with overdoses of isoflurane until the rats underwent cardiac arrest.

### Morphometric analyses

After euthanasia, animals were weighed (weight expressed in g). The abdominal cavity was reopened, and the remaining bowel was harvested from the duodenum to the rectum. Therefore, the small bowel diameter was measured at three different points (proximal, medium and distal points, expressed in mm), and the total length from the end of duodenum to the anastomosis was measured (expressed in cm). The diameter of the remaining colon was measured at the midpoint of the length from the anastomosis to the anus. In the control group, the entire small bowel and colon were harvested separately.

For all animals, a ratio of intestinal length to body weight was calculated and used for comparisons among groups.

### Histopathological evaluation

For microscopy assessment, the tissue specimens were immediately fixed in buffered formalin (pH 7.4) for 24 hours, dehydrated, and then embedded in paraffin wax. Five-micrometer-thick sections were taken, deparaffinized and routinely stained with hematoxylin and eosin (HE). Prepared samples were evaluated by a single pathologist blinded to the study groups of samples.

The following parameters were evaluated: villus height, crypt depth, and thickness of the lamina propria and muscular layer. Evaluations were performed under an Olympus CX 41 light microscope (Shinjuko, Tokyo, Japan) at 40x magnification using an ocular micrometer. Images obtained from an Olympus DP 20 high-definition photo receiver attached to the light microscope were interpreted. Villus height and crypt depth were calculated as the mean of 10 villi and 10 crypts and were measured in µm. Final values in the comparisons between groups were relative to the intestinal wall.

### Total RNA isolation and reverse transcription

Total RNA was isolated from all small bowel and colon samples as previously described 12.

The complementary DNA (cDNA) samples were synthesized from total RNA samples (2.0 μg) with 200 U of SuperScript III RNase H-RT (Invitrogen). Reverse transcription was carried out at 42°C for 50 minutes using oligo(dT)s as primers. The resulting cDNA solution was stored at −20°C.

### Molecular analysis

Molecular analysis consisted of studying the expression of Bcl-xl and Bax genes with RT-PCR according to previous descriptions from our laboratory 16,17. β-actin was used as a housekeeping gene.

### Statistical analysis

The results were reported as the mean±SEM for parametric data and as the median (min-max values) for nonparametric data. Statistical analysis was performed by t-test and analysis of variance with a multiple comparison test (Bonferroni multiple t-test) for parametric data and Mann-Whitney or Kruskal-Wallis test for nonparametric data. The level of significance was *p*≤0.05.

## RESULTS

### Body weight and morphometric analysis

It was verified that young rats presented weight gain during the experiments after enterectomy (42.4±21.5), whereas adult rats presented weight loss during the experiment (70.4±26.0, *p*<0.0001). All other measurements demonstrated significant differences between young animals and adults for both periods after enterectomy (Table 1).

### Histomorphometric analysis

**Figure 1:**
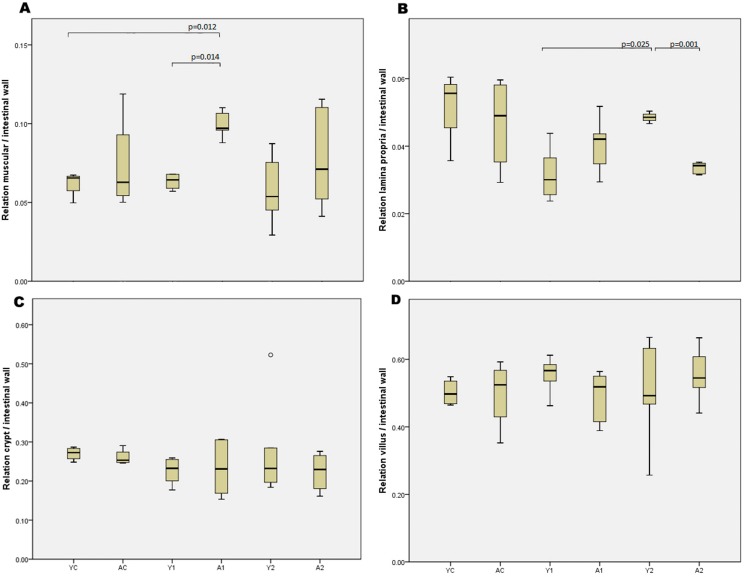
Results of small bowel histomorphometric analysis.

**Figure 2:**
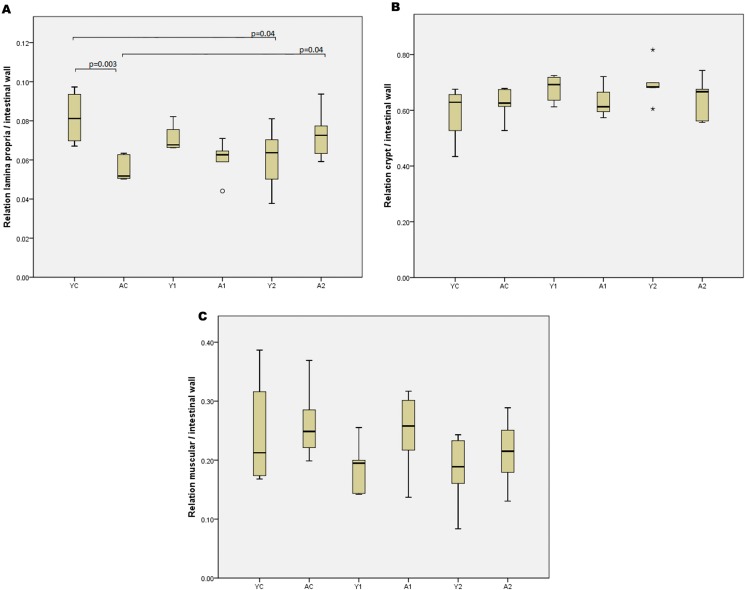
Results of colon histomorphometric analysis.

**Figure 3:**
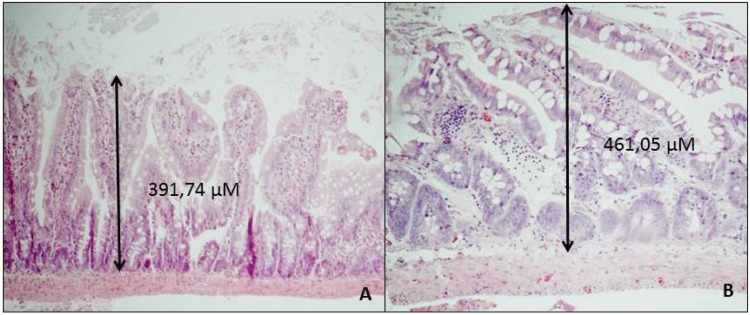
Histological findings in the remaining small bowel 21 days after enterectomy (A – young animal; B – adult animal). Note that villus heights relative to the thickness of the intestinal wall are similar (HE, magnification 40X).

All histomorphometric data for the small bowel and colon in the study groups are expressed in Figures 1 and 2, respectively. Comparisons with the control animals demonstrated that enterectomy did not promote any alterations in the histomorphometry of the remaining small bowel in weaning or adult animals. However, the thickness of the lamina propria was larger in weaning animals than in adults 21 days after enterectomy (*p*=0.001). There were no significant differences in the other histomorphometric parameters (Figure 3). Regarding colon histomorphometric analysis, it was verified that the thickness of the lamina propria was larger in control young animals than in control adults (*p*=0.003). Enterectomy increased the thickness of the lamina propria after 21 days in the adult animals (*p*=0.04) but decreased thickness of the lamina propria in young animals (*p*=0.04).

### Molecular analysis

**Figure 4:**
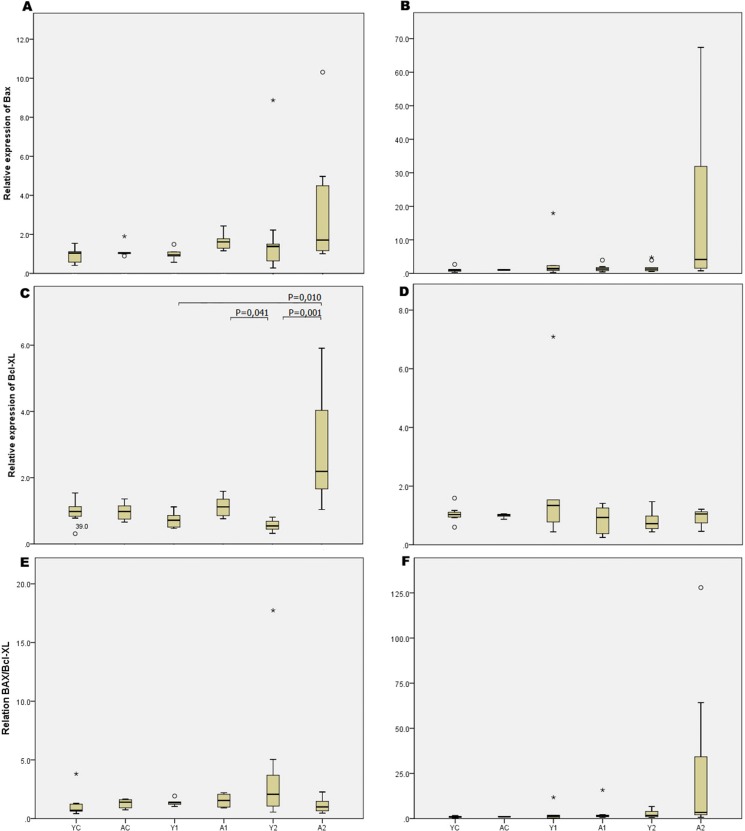
Results of molecular analysis (A, C, E - small bowel; B, D, F – colon).

The molecular studies showed that enterectomy promoted increased expression of the Bcl-xl gene in the small bowel of adult animals compared with that in growing animals. However, comparisons of the ratio between Bcl-xl and Bax expression showed no differences among groups. The gene expression values of the different groups are detailed in Figure 4.

## DISCUSSION

Although the incidence of SBS is higher in infants than in adults, few published studies have used growing animals. There are several difficulties associated with operating on and maintaining young and weaning rats because of the friability and size of their tissues, their bowel caliber, their erratic response to anesthetic drugs and their lower resistance to dehydration after massive enterectomy than adult rats. In fact, the mortality of animals after the procedure was considerable in our experiment despite pilot procedures and technically trained surgeons.

Most rodent models of massive bowel resection create SBS by ligating mesenteric branches and resecting most of the small bowel from a few centimeters after the duodenum to a few centimeters before the cecum 7. In the current investigation, our objective was to create a rat model of SBS that most closely resembled the human pediatric condition in which the distal ileum, ileocecal valve and proximal colon are resected due to necrosis 15. Normal rat anatomy is similar to human intestinal malrotation, and the whole intestine is surrounded by the superior mesenteric arterial and venous trunk and its branches. Therefore, we observed that ligation of the superior mesenteric vessels after the fifth branches was responsible for nearly 60% ischemia in the distal small bowel, cecum and proximal colon, which is the same segment that is impaired in pediatric SBS. Thus, only one vascular ligation was performed, reducing the time of surgery and the manipulation of extremely delicate mesentery.

In pilot animals, it was observed that some animals survived to the anesthetic and operation procedures but died in the first postoperative days. After this pilot study, we opted for 50% glucose and sodium chloride supplementation in the water offered ad libitum to operated animals in all groups, and we observed that the incidence of mortality decreased.

Current practice shows that children usually have a better prognosis after massive bowel resection that adult patients. In adults, adaptation reaches a plateau after approximately 1 year, and further changes to adaptive function are unlikely. The adaptation process seems to be more effective and last longer in children than in adults, allowing children to sometimes be weaned from parenteral nutrition several years after their initial resection 18. The findings of the present study seem to corroborate this fact as weaning rats presented significant weight gain from the 4^th^ to 21^st^ day after massive enterectomy, whereas adult animals continued to lose weight even 21 days after surgery. Therefore, the adaptative changes in the remnant bowel truly appeared to be more efficient and faster in growing animals than in adults.

In fact, one of the most remarkable results of the present study was the significantly higher relative length and diameter of the residual small bowel and colon in weaning rats than in adults after resection of the same percentage of their intestines. Although it is not mentioned or valorized in most clinical and experimental works, colonic elongation may have an important role in the adaptation processes of hydroelectrolytic control and nutritional recuperation.

An important adaptive response following massive small bowel resection is characterized by increased crypt cell proliferation, resulting in deepened crypts and increased villus height 19. In the current work, we aimed to study whether there were any differences in these histomorphological aspects between growing and adult SBS animals. In addition, we analyzed other bowel layers, which are not usually evaluated in most intestinal adaptation experimental and clinical studies.

Initially, we could not detect any differences in the villus height or crypt depth between adult and young rats in either period of study. Based on these data and the results of morphometric analysis (Table 1), we may conclude that among the mechanisms of adaptation after massive intestinal resection, the elongation and dilatation of the residual bowel observed in weaning rats were more important mechanisms of adaptation than increased villus height.

Among the structural components of the intestinal wall, the lamina propria is composed of a connective tissue between the epithelium and the muscularis mucosae 20. This connective tissue is very loose and contains a variable population of cells, including fibroblasts, lymphocytes, plasma cells, macrophages, eosinophilic leukocytes, and mast cells. It provides support and nutrition to the epithelium and binds underlying tissue 21. Current evidence supports that mesenchymal cells from the lamina propria closely interact with the epithelium from an early period of development until adult life; they are involved in intestinal stem cell niche function, produce factors regulating epithelial proliferation and differentiation, and actively support epithelial homeostasis upon damage 20.

In the present investigation, we observed that 21 days after enterectomy, the lamina propria was thicker in young animals than in adults (*p*=0.001). As in all physiologic processes, intestinal adaptation is faster in rats than in humans, lasting nearly 14-21 days in rats and 1 year or more in humans. It is known that in children with SBS, the mechanisms of intestinal adaptation are more efficient and last longer than those in adults 14,15,19. A possible explanation for this difference is based on the current results that the enlarged lamina propria may provide nutrition to enterocytes to meet increased proliferation and metabolic demands.

Regarding the dynamics of cells in the intestinal mucosa, it is known that regulation of the balance between crypt cell proliferation and programmed cell death is a normal process required for maintaining the normal crypt-villus axis. The crypts maintain normal stem cell numbers by eliminating damaged cells through the mechanism of apoptosis. Experimental studies have shown that crypt apoptosis increases early postintestinal resection 21,22 and that this increase persists for at least one week postoperatively and includes the expression of proapoptotic genes. Bax is one of several highly expressed proapoptotic proteins that plays an important role in regulating intestinal crypt apoptosis.

Our results from the molecular studies showed that the antiapoptotic Bcl-xl gene was more highly expressed in the adults than in the growing rats. However, we did not observe any significant differences when we studied the relation between the expression of antiapoptotic and proapoptotic genes. We may conclude that other genes may be involved in the complex mechanisms of intestinal adaptation after massive intestinal resection, which is similar to what occurs other regenerative processes, such as liver regeneration, as we demonstrated that no changes in the expression of the Bcl-xl gene were observed in the remaining liver parenchyma in a model of massive hepatic resection 23.

In conclusion, the present work presented a feasible and reliable model of pediatric SBS and showed that there are morphometric, histological and molecular differences in the adaptation processes of growing and mature organisms. These data may facilitate and promote further studies aimed at understanding and treating children and adults with SBS.

## AUTHOR CONTRIBUTIONS

Tannuri AC and Rotondo IG conceived and designed the study, and were responsible for data interpretation and manuscript drafting. Barros GG, Vaisberg VV, Mendes-Neto C, Paes VR, Coelho MC, Gonçalves J and Serafini S were responsible for data acquisition and analysis. Tannuri U was responsible for the data final interpretation and critical revision.

## Figures and Tables

**Table 1:** Morphometric data.

	Y1	A1	P (Y1xA1)	Y2	A2	P (Y2xA2)
Small bowel length/body weight	0.54±0.15	0.22±0.05	0.002	0.40±0.07	0.30±0.06	0.001
Colon length/body weight	0.12±0.02	0.05±0.02	<0.0001	0.10±0.02	0.08±0.02	0.1
Proximal small bowel diameter/body weight	0.0064±0.002	0.0021±0.0004	<0.0001	0.0054±0.002	0.0038±0.002	0.2
Medium small bowel diameter/body weight	0.0064±0.001	0.0023±0.0007	<0.0001	0.004±0.0013	0.002±0.0005	0.01
Distal small bowel diameter/body weight	0.01±0.002	0.004±0.001	<0.0001	0.009±0.005	0.007±0.003	0.4
Colon diameter/body weight	0.009±0.004	0.0037±0.001	0.001	0.0073±0.001	0.0043±0.001	0.0005
